# The views of people using homeless services about learning disability

**DOI:** 10.1177/17446295231215412

**Published:** 2023-11-10

**Authors:** Karen McKenzie, Ruth Robson, George Murray, Matt Kaczmar, Dale Metcalfe, Alex Shirley

**Affiliations:** Department of Psychology, 5995 Northumbria University, UK; Evaluation, Learning & Research Manager, Changing Lives, UK; Clinical Psychologist, GCM Records LLP, UK; GP, Cruddas Park Surgery, UK; Northumbria University, UK; Director of Development, Changing Lives, UK

**Keywords:** early identification, homelessness, knowledge, learning disability

## Abstract

**Aims:**

People with a learning disability are at increased risk of becoming homeless, but little is known about how learning disability is viewed by people accessing homeless services. This study aimed to obtain the views of people experiencing homelessness about learning disability, in the context of a project that was exploring how to increase identification of learning disability.

**Methods:**

A qualitative approach was used, and 19 adults were interviewed who were receiving support from homeless services in the North-East of England. Information from the interviews was analysed using thematic analysis.

**Results:**

Four themes were identified relating to understanding of learning disability, the role of identification, day-to-day challenges, and experiences of services.

**Conclusion:**

There is a need to: promote better understanding of learning disability; for early identification processes that involve the person in a meaningful way; and the provision of support that is non-stigmatising, practical and which addresses health concerns.

## Introduction

Intellectual disability is the internationally used term to describe people who meet the criteria of having significant difficulties with their cognitive functioning and their adaptive skills. These difficulties must have been present before adulthood (American Psychiatric Association, 2013; World Health Organisation, 2022). In the United Kingdom (UK), terms that are commonly used in place of intellectual disability, are ‘learning disability’ and ‘learning difficulty’ (see Cluley, 2018). Somewhat confusingly, both learning disability and learning difficulty are used in other countries and contexts to describe people who have specific educational needs, such as those with dyslexia (e.g., NHS Data Model and Dictionary, 2022).

Having a learning disability increases the risk of experiencing homelessness compared to those without a learning disability (Brown and McCann, 2021; Durbin et al., 2018). While the current prevalence rate of learning disability within homeless services in the UK is unknown, the rates from previous studies have been estimated to be between 12 and 28% (Bremner et al., 1996; Oakes and Davies, 2008). Internationally, including the UK studies, prevalence rates have been found to range between 12 and 39% (Durbin et al., 2018). While the methods of establishing prevalence vary and often rely on self-report (Brown and McCann, 2021), the figures suggest that a high proportion of those who experience homelessness are also likely to meet the criteria for learning disability.

The increased risk for experiencing homelessness has been attributed to multiple and often co-occurring factors. A review by Brown and McCann (2021) identified the most notable as being poor mental health, low levels of educational attainment, alcohol dependence and other substance misuse, and the breakdown of family and support relationships. These results suggest the need for support from health, education, and other services at an early stage to help minimise the risk of homelessness. Indeed, a key aim of the recent Homelessness Reduction Act 2017 (https://www.legislation.gov.uk/ukpga/2017/13/contents/enacted) was to ensure that health and local authority services worked collaboratively to prevent homelessness occurring (Paudyal and Saunders, 2018).

When homelessness does occur, it can be extremely detrimental for those experiencing it. People with a learning disability already face poorer health and increased mortality compared with their peers who do not have a learning disability (The LeDeR Team, 2021) and being homeless can further contribute to poor physical and mental health (Plage and Parcell, 2022). In particular, it leaves people with a learning disability extremely vulnerable to financial, sexual and physical exploitation and abuse (Lougheed and Farrell, 2013). Perhaps because of this vulnerability, people with a learning disability who are homeless may try to hide their difficulties. They may also be unaware that they would be considered to have a learning disability because they have not previously been identified as such or because they have not fully understood what their diagnosis means. Emerson and Glover (2012) note that over 50% of adults with a learning disability are ‘hidden’ i.e., their learning disability is not known to, or recorded by, services.

This means, however, that people with a learning disability who are homeless may not receive the support they need to navigate complex health, social care, housing, and benefits systems (McKenzie et al., 2019a). Previous research has indicated that staff in homeless services may also lack knowledge about learning disability which would help them recognise that an individual may need additional support. There is, however, only limited research about the knowledge and experience of learning disability of those using homeless services (McKenzie et al., 2019b). The aim of the present study was to explore the views of people using homeless services about learning disability. This was in the context of a wider study that was exploring ways to improve the identification and support of people with a learning disability in people who were experiencing homelessness, through the use of a learning disability screening questionnaire and strengthening links with primary care services (McKenzie et al., 2023). Recent guidance has highlighted the importance of involving service users in the wider design of services (e.g., NHS England, 2022a). Obtaining the views of current users of homeless services was, therefore, considered to be important in shedding light on how learning disability was understood by them, their own experiences (if any) of identification and support and their perspectives on important factors to consider when aiming to improve identification and support of people with a learning disability.

## Method

### Design and ethics

The study used a qualitative design and received ethical approval from the first author’s university ethics committee. Thematic analysis (Braun and Clarke, 2006) was used to code data obtained from semi-structured interviews.

### Study Context

The study formed one aspect of a wider project which was exploring the use and impact of a screening tool as a means of helping to identify and facilitate support for people with a previously unidentified learning disability, who were using the homeless service.

### Participants

Participants were recruited using purposive sampling from people using a homeless service in the North-East of England. Additional inclusion criteria were that the person was aged 18 years or older and had provided their own informed consent, which was recorded in an online consent form. A total of 19 people took part (see Table 1 for demographic information). Of the 19 participants, nine reported having a learning difficulty (a term which is frequently used interchangeably to describe learning disability), two reported having a learning disability, one had experienced a brain injury, and three experienced mental health difficulties. Participants are identified by number in the text to ensure anonymity.

**Table 1.** Participant information.

Participant Number	Age	Gender
1	49	Male
2	26	Male
3	36	Female
4	27	Male
5	53	Female
6	32	Male
7	40	Female
8	32	Male
9	30	Male
10	55	Male
11	35	Female
12	35	Male
13	41	Male
14	42	Male
15	25	Male
16	22	Male
17	36	Female
18	46	Male
19	60	Male

### Procedure

Potential participants were provided with information about the study by staff at the homeless service. An accessible participant information sheet was available, and support was offered by staff to read the information, if needed. All participants had an opportunity to ask questions about the project. Those who provided consent to take part were invited to give their views in a short, semi-structured interview. This was conducted by an experienced researcher within the homeless service who did not have a role in the direct support of the participants. The interview asked about the person’s understanding of learning disability, their experiences (if any) of it and the factors they thought were important in identifying and supporting people with a learning disability. Interviews lasted between 5-20 minutes.

As some participants did not want their interview to be recorded, the decision was made not to digitally record any of the interviews to ensure consistency across participants. Instead, a researcher took notes throughout the interview and noted verbatim particular phrases that seemed to capture a participant’s view well. Only these verbatim phrases were used as quotes in the results section to illustrate the general points that were made. Participants were provided with a debrief at the end of the study, which included information about how to withdraw their data at a later date if they wished to do so.

### Analysis

The notes from the interviews were analysed using thematic analysis by the first author, who is a clinical psychologist and researcher with many years of experience of working with people with a learning disability. The interview notes for each individual participant were first read on a number of occasions to ensure the content became familiar. Initial sections of texts were then coded together under provisional headings. These were then developed into consistent strands, which were then organised under coherent themes. The final themes were then shared with the wider research team to ensure they were consistent with the interview content.

## Results

The analysis indicated four main themes as outlined below.

### Theme 1: ‘*Don’t know when it is a difficulty or disability’: Understanding of learning disability*

The first theme related to the participants’ limited understanding of learning disability, particularly in the context of other terminology that is commonly used to describe academic or other learning difficulties. There was some confusion between the terms ‘learning disability’ and ‘learning difficulty’: ‘*Don’t know when it is a difficulty or disability. I wouldn’t really use these terms’* (P1), although there was a sense that they referred to cognitive difficulties: *‘…means people struggle with reading and writing and have difficultly learning things’* (P3). Learning disability was thought to refer to a wide range of issues, including specific learning difficulties and behaviour: *‘things like dyslexia… and dyspraxia…there are a lot of things you can add to it, behaviour stuff, emotional stuff’* (P19).

The confusion about terminology was compounded by the early experiences of some participants. P19 described that he was thought to have dyslexia but was sent to a school for people with learning disabilities:

I was there for 2 years when I was 11 and I must have caught up on about 5/6 years of schooling. I went back to mainstream school, but I started struggling again and I went back to [name of school] when I was about 14.5 years.

The understanding of different conditions was frequently referred to in the context of the person’s own experience: *‘…probably something to do with the brain. I had a brain injury when I was in junior school’* (P5), or that of someone they knew: ‘*My brother had learning disabilities, he had to go to a different school’* (P6) and *‘My knowledge of this is from my sister who grew up in care, she has always had problems with learning, she didn’t go to mainstream school’* (P1).

In addition, many participants contrasted ‘learning disability’ with conditions that they had been diagnosed with. For example, P6 had a diagnosis of ADHD:

I had all this energy bursting to get out, I couldn’t sit still, I couldn’t focus on anything, I couldn’t concentrate. I would constantly get into trouble, but that would wind me up, it wasn’t like I had a choice to be anything different, I couldn’t help being me. (P6).

In other cases, participants attributed their performance at school to their immaturity and behaviour at that time, rather than to a developmental difficulty:

I’ve defo not got any learning disabilities or differences. School was easy for me. I was daft in school and I didn’t try and I only scraped C’s but I’ve never had any issues with learning or taking stuff in, was just a dickhead that messed about (P2).

### Theme 2: ‘*I don’t think about it as a disability, it is a hindrance’*: the role of identification

The second theme relates to the role of identification and of making sure that those undergoing assessment fully understand the process.

Most participants had limited information about assessment and diagnostic processes and felt that professionals had not taken the time to explain these to them: ‘*Nobody ever said anything about what the tests were for or what the results were*’ (P7)**.** Many were also unsure if they had ever received a formal diagnosis for their difficulties and if they had, what that diagnosis was. P8 thought he: ‘…*may have dyslexia, although this has never been diagnosed.*’

The delay in receiving a diagnosis was viewed as having had a negative impact on the lives of many of the participants. P19 was unsure if he has ever had a formal diagnosis but recalls reading something about him having a learning disability in the past. The lack of clarity led him to view his condition very negatively: *‘I don’t think about it as a disability, it is a hindrance*.’

A few participants had received a formal diagnosis (although not for learning disability). For P9, who was diagnosed with dyslexia as a child, this resulted in more one-to-one support at school. P6’s diagnosis of ADHD enabled her to *‘…understand why I was how I was, and why I got frustrated*.’ Contributor P4 felt his diagnosis of ADHD had helped, as previously he was labelled as the ‘*naughty kid*’. Likewise, P19 reported that his diagnosis was helpful, although his difficulties weren’t picked up until he was in his twenties and in prison.

I didn’t learn to read until I was 24, it was in Prison, I came out with a City and Guilds. All the way through school I was told I was naughty, but I couldn’t sit in a classroom and concentrate. I was sent to an approved school, I even got sent to a boarding school cause I was that bad, I missed a full 2 years schooling. I was dead chuffed when I got my education and made something for mesell in prison. Does make you think, if they had found out when I was at school that I had dyslexia how things could have been different”

When asked about the possibility of receiving a diagnosis at this stage in their lives, some were ambivalent: ‘*wouldn’t be bothered either way* ‘(P6), others felt it wouldn’t apply to them, but if it did, they would have to ‘*get on with it’* (P7). One person felt it would be too late for them: ‘*I’m too old for it now, no point in getting a diagnosis, won’t make any difference now’* (P10).

### Theme 3: ‘I can read owt but I don’t always understand what it means’: Day to day challenges

The third theme explored the day-to-day difficulties that the participants experienced, both in the past and at the time of the study. These included challenges with comprehension: ‘*people can explain things, but it doesn’t really seem to go in, me and English just doesn’t seem to work’* (P6), and literacy skills: ‘*I can read owt but I don’t always understand what it means. My writing is neat and tidy, but I can’t spell very well’* (P11) and ‘*I’m a neat writer but my spelling isn’t great’* (P12).

Such difficulties had practical consequences: ‘*Any forms to fill in, I need to take it away and I have to read it 3 or 4 times before I understand it*’ (P19). There were also negative consequences for relationships with others: *‘I lose concentration, get bored easily…I just can’t be bothered with certain people either*’ (P10). This could result in feelings of social isolation: ‘*I’ve struggled with relationships and I don’t have friends*’ (P19)

Many participants relied on others for help with day-to-day tasks. P9 struggled with writing letters and relied on predictive text to help him when writing. He also needed help from others with things like job searching. P19 has tried to made adaptations: ‘*If I’m writing, I avoid words I don’t know how to spell and try and think of easier words to use instead*.’ Likewise, P13 used apps to help with his writing.

### Theme 4: ‘*What do you want me to do about it*?’ Experiences of services

The final theme outlines some of the experiences that participants had of support services. Most participants felt that the support that they had received from education services as children had been unhelpful and stigmatising: ‘*Teachers said I was stupid, thick… when I went to mainstream school, they put me in what they called the dunces class, that’s how they referred to us’* (P19). This negative approach could result in behavioural difficulties. P14 described himself as a ‘*special needs, naughty boy*’ and noted that his ‘*generation weren’t understood*.’ P8 was: ‘*labelled as a workie ticket*,’ with an associated negative impact on behaviour: ‘*always getting into bother*.’ P10 was expelled from school aged 12 and P15 noted: ‘*I was badly behaved, but I used to get really stressed when they used to give me work, I wasn’t interested and I couldn’t understand anything*.’

For others, their behaviour resulted in them being placed in a ‘special class’: ‘*It was mainly for behaviour but down to me learning too*’ (P16), which led to further isolation from the other children: ‘*We’d get called all sorts for being in that class’* (P7).

Some participants had also experienced negative or limited support from health services as adults: ‘*I did go to the doctors once, but I got agitated with him, he said “what do you want me to do about it?”* (P14). Similarly, P17 was unsure if she was on the disability register, and reported feeling as if, since leaving school, she has had no support for her conditions and mental health. Similarly, P11, believed she was on the learning disability register, as she received an allowance for her disabilities, but reported that her diagnosis didn’t seem to trigger any additional support, such as annual health checks.

## Discussion

The study aimed to explore the views of people experiencing homelessness about learning disability. This was in the wider context of a project which was evaluating the introduction of a screening tool for intellectual disability into the homeless services in order to improve identification and support of people with a learning disability (McKenzie et al., 2023). Overall, the study found that there was limited understanding about the term ‘learning disability’ and it was often understood in the context of the person’s own experiences or those of someone they knew, or was referred to using a different term – such as ‘learning difficulty.’ Previous research has found that there is also limited knowledge about learning disability among some service providers and professionals (McKenzie et al., 2022; Whittle et al., 2018).

This limited understanding is perhaps partly a result of changes in terminology over time, as the purpose of classification alters, for example to identify those who needed economic support, education and training or institutional health care (Murray and McKenzie, 2020). Even within the same time-period, the meaning of the terms can differ between countries and between services within the same country (Cluley, 2018), which can potentially add to the confusion.

In the present study, many participants preferred to refer to themselves as having a learning difficulty. In some cases, this term may have been appropriate because their difficulties appeared to be specific e.g., with reading and writing, rather than the global and significant developmental difficulties in adaptive and cognitive functioning which are defining of learning disability (American Psychiatric Association, 2013; World Health Organisation, 2022). For others, denial of their learning disability may have arisen because of shame or a fear of being victimised (McKenzie et al., 2019b). This is perhaps unsurprising, given that some participants described a process whereby they were ridiculed and stigmatised, most often at school, as a result of their difficulties. Many attributed their subsequent behaviour to these experiences, which they felt had had a long-lasting negative impact on their lives. The importance of receiving support that was helpful and not stigmatising, particularly at a young age, was stressed.

There was also a clear need for support in adulthood. The participants described a range of challenges that they experienced in their daily lives, such as limited understanding of information, poor social relationships, and difficulty reading and completing forms. They often relied informally on others for support to help them deal with these challenges. The experience of more formal support from health services was not always positive and a few participants felt that their health issues had not being properly addressed or had even being dismissed. It is well documented that people with a learning disability face significant health inequalities (The LeDeR Team, 2021), and that experiencing homelessness can exacerbate physical and mental health issues (Plage and Parcell, 2022). If a person’s learning disability is not known to services, then they will not be offered reasonable adjustments in their health care, such as annual health checks.

The role of early identification of conditions was highlighted, although most of the participants did not experience this themselves and some felt that their difficulties had still to be formally recognised. This is a common experience, with Emerson and Glover (2012) noting that over half of adults may be unknown to services as having a learning disability. This may be because they were not identified as meeting the criteria in childhood or because this information is lost when they transition from child to adult services. Other participants had undergone an assessment and diagnostic process but were left uncertain about what their diagnosis was and what it meant for them. The need to involve the person in the process was emphasised - to ensure that they understood what would be involved, why it was taking place, as well as being informed of the outcome. While the importance of empowering people with a learning disability to make choices about their own health care, as well as becoming involved in the wider design of services has been emphasised in health service guidance (e.g., NHS England, 2022a), the results from the study suggest that people do not always experience this empowerment in practice.

### Implications for practice

Consistent with a previous, small study (McKenzie et al., 2019a), the results of the present study have a number of implications for practice. Learning disability staff can use their expertise to educate and inform other health and third sector services about learning disability, with the aim of improving identification, understanding and support. This may include signposting to evidence-based learning disability screening tools that can be used by those with both a specialist and non-specialist background to help identify people who are likely to meet the criteria for learning disability.

Learning disability staff also have an important role in signposting services to relevant resources, as well as supporting their use in practice. This might include increasing the empowerment of people with a learning disability in health service contexts, by setting up meetings with local advocacy groups, ensuring that information about processes and systems are accessible and employing people with a learning disability, for example as service quality checkers (NHS England, 2022b). There are also resources that are specifically developed for service providers for people with a learning disability who experience homelessness, such as the Learning Disability and Homelessness Toolkit (Tickle et al., 2022).

Learning disability staff can also provide advice about local service provision and processes, such as being placed on the GP learning disability register and receiving the associated reasonable adjustments in health care, such as annual health checks. Indeed, research suggests that an important factor in providing the best primary care services to people experiencing homelessness was having nurses as members of the team (Jego et al., 2018).

### Limitations

The study had some limitations. The participants self-reported if they had any conditions, but this was not independently assessed. It was not, however, an inclusion criterion that participants had been diagnosed for learning disability, as the aim of the study was to obtain the views of people who were experiencing homelessness about learning disability, regardless of whether they considered themselves to have a learning disability or not. Notes were taken about the interview at the time they took place, instead of recording them. It may be that some important points were missed as a result. The study was conducted in a homeless service in the North-East of England and the results may not be generalisable to other services or geographical areas.

### Conclusion

The aim of the study was to explore the views of people who were experiencing homelessness about learning disability, in the context of a project that aimed to improve identification of learning disability. The results suggested that knowledge about learning disability is somewhat limited and there is some confusion about the different terms that are used. The role of early identification and meaningful involvement in the process were both highlighted as important. Subsequent support, both formal and informal, should be non-stigmatising, address practical difficulties that people experience, and provide access to appropriate healthcare.

## References

[bibr1-17446295231215412] American Psychiatric Association (2013) Diagnostic and Statistical Manual of Mental Disorders (5th ed.*)* Washington, DC: American Psychiatric Association.

[bibr2-17446295231215412] Braun V Clarke V (2006) Using thematic analysis in psychology. Qualitative Research in Psychology 3(2): 77-101.

[bibr3-17446295231215412] Bremner AJ Duke PJ Nelson HE et al. (1996) Cognitive function and duration of rooflessness in entrants to a hostel for homeless men. British Journal of Psychiatry 169: 434-439. 10.1192/bjp.169.4.434 8894193

[bibr4-17446295231215412] Brown M McCann E (2021) Homelessness and people with intellectual disabilities: A systematic review of the international research evidence. Journal of Applied Research in Intellectual Disabilities 34(2): 390-401. 32959955 10.1111/jar.12815

[bibr5-17446295231215412] Cluley V (2018) From “Learning disability to intellectual disability”—Perceptions of the increasing use of the term “intellectual disability” in learning disability policy, research and practice. British Journal of Learning Disabilities 46(1): 24-32.

[bibr6-17446295231215412] Durbin A Isaacs B Mauer-Vakil D et al. (2018) Intellectual disability and homelessness: A synthesis of the literature and discussion of how supportive housing can support wellness for people with intellectual disability. Current Developmental Disorders Reports 5(3): 125–131.

[bibr7-17446295231215412] Emerson E Glover G (2012) The ‘transition cliff’ in the administrative prevalence of learning disabilities in England. Tizard Learning Disability Review 17(3): 139-143.

[bibr8-17446295231215412] Jego M Abcaya J Ștefan D et al. (2018) Improving health care management in primary care for homeless people: a literature review. International Journal of Environmental Research and Public Health 15(2): e309. 10.3390/ijerph15020309 PMC5858378 29439403

[bibr9-17446295231215412] Lougheed DC Farrell S (2013) The challenge of a “Triple Diagnosis”: Identifying and serving homeless Canadian adults with a dual diagnosis. Journal of Policy and Practice in Intellectual Disabilities 10: 230–235.

[bibr10-17446295231215412] McKenzie K Murray GC Martin R et al. (2022) Knowledge of social care staff about learning disability: Twenty years on. Learning Disability Practice Online ahead of print. doi: 10.7748/ldp.2022.e2182

[bibr11-17446295231215412] McKenzie K Murray GC Metcalfe D et al. (2023) Using the Learning Disability Screening Questionnaire to help identify people with an intellectual disability in homeless services. Journal of Applied Research in Intellectual Disabilities. Online ahead of print. doi: 10.1111/jar.13150 37635318

[bibr12-17446295231215412] McKenzie K Murray GC Wilson H et al. (2019a) Homelessness – ‘It will crumble men’: the views of staff and service users about facilitating the identification and support of people with an intellectual disability in homeless services. Health and Social Care in the Community 27(4): e514-e521. 30983058 10.1111/hsc.12750

[bibr13-17446295231215412] McKenzie K Murray GC Wilson H et al. (2019b) A tool to help identify learning disabilities in homeless people. Nursing Times 115(8): 26-28.

[bibr14-17446295231215412] Murray GC McKenzie K (2020) Making ‘Imbeciles’ of the Poor: A Study of the Lives of People in the Berwick-upon-Tweed Union Workhouse categorised as ‘Imbeciles’ in the 1881 Census*.* Berwick upon Tweed, England: GCM Records.

[bibr15-17446295231215412] NHS Data Model and Dictionary (2022) Learning Difficulty. Available from: https://www.datadictionary.nhs.uk/nhs_business_definitions/learning_difficulty.html#:∼:text=aPERSONPROPERTY.-,ALearningDifficultyisatypeofSpecialEducationNeeds,toread%2Cwrite%2Cspelletc

[bibr16-17446295231215412] NHS England (2022a) Why is it important to involve people*.* Available from: https://www.england.nhs.uk/learning-disabilities/about/get-involved/involving-people/why-is-it-important-to-involve-people/

[bibr17-17446295231215412] NHS England (2022b) Involving people with a learning disability*.* Available from: https://www.england.nhs.uk/learning-disabilities/about/get-involved/involving-people/involving-people-with-a-learning-disability/

[bibr18-17446295231215412] Oakes PM Davies RC (2008) Intellectual disability in homeless adults: A prevalence study. Journal of Intellectual Disabilities 12: 325–334. 19074937 10.1177/1744629508100496

[bibr19-17446295231215412] Paudyal V Saunders K (2018) Homeless Reduction Act in England: impact on health services. Lancet 392(10143): 195-197. 30043743 10.1016/S0140-6736(18)31383-7

[bibr20-17446295231215412] Plage S Parcell C (2022) Access to health for people experiencing homelessness. European Journal of Homelessness 16(1): 29-52.

[bibr21-17446295231215412] The LeDeR Team (2021) The Learning Disabilities Mortality Review (LeDeR) Programme Annual Report 2020. Bristol: The University of Bristol.

[bibr22-17446295231215412] Tickle A Aseervatham V Andrews L et al. (2022) Learning Disabilities and Homelessness Toolkit*.* Available from: https://homeless.org.uk/knowledge-hub/learning-disabilities-and-homelessness-toolkit/

[bibr23-17446295231215412] Whittle EL Fisher KR Reppermund S et al. (2018) Barriers and enablers to accessing mental health services for people with intellectual disability: A scoping review. Journal of Mental Health Research in Intellectual Disabilities 11(1): 69-102.

[bibr24-17446295231215412] World Health Organization . (2022). ICD-11: International Classification of Diseases (11th revision). https://icd.who.int/

